# Transcriptome Analysis of *Gerbera hybrida* Ray Florets: Putative Genes Associated with Gibberellin Metabolism and Signal Transduction

**DOI:** 10.1371/journal.pone.0057715

**Published:** 2013-03-05

**Authors:** Qi Kuang, Lingfei Li, Jianzong Peng, Shulan Sun, Xiaojing Wang

**Affiliations:** Guangdong Provincial Key Lab of Biotechnology for Plant Development, College of Life Sciences, South China Normal University, Guangzhou, China; Centro de Investigación y de Estudios Avanzados del IPN, Mexico

## Abstract

In this study, the transcriptome of the *Gerbera hybrida* ray floret was constructed using a high-throughput Illumina sequencing platform. All 47,104 UniGenes with an average length of 845 nt and an N50 equaling 1321 nt were generated from 72,688,546 total primary reads after filtering and assembly. A total of 36,693 transcripts were annotated by comparison with non-redundant National Center for Biotechnology Information (NCBI) protein (Nr), non-redundant NCBI nucleotide (Nt), Gene Ontology (GO), and Kyoto Encyclopedia of Genes and Genomes (KEGG) databases after removing exogenous contaminated sequences. The majority of the genes that are associated with gibberellin metabolism and signal transduction were identified. The targets for signal transduction of other plant hormones were also enumerated. Our study provides a systematic overview of the hormone signal transduction genes that are involved in ray floral development in Asteraceae and should facilitate further understanding of the crucial roles of phytohormones in plant growth.

## Introduction

Unlike species with single type of flowers, *Gerbera hybrida*, a unique Asteraceae plant with three special types of florets that are condensed at the same receptacle, is an important model for studying flower growth [1], [2], [3]. Its marginal ray florets and trans florets are female with non-functioning staminodes, while the inner disc florets are hermaphrodites. Notably, the ray florets with aborted stamens have long ligulate petals, whereas the disc florets, which contain fertile pollens, merely have short degenerate petals. Previous studies have found that the changes to the stamens and pistils in *G. hybrida* occur throughout the mid-developmental stages instead of during the flower primordia stages [4]. One reasonable hypothesis for this phenomenon is that the fertile switching of the *G. hybrida* florets’ sexual organs may be manipulated by endogenous phytohormones. The crucial roles of various phytohormones in flower development have been investigated in many studies. For example, Jasmonate (JA) influences stamen development [5], [6], Auxin 2,4-D can reactivate the pistillodes in the staminate flower of date palm, and other hormones can reverse the staminode in the pistillate flower to form fertile stamens [7]. Among these elements, a key hormone in floral opening is gibberellin (GA). In a GA-deficient mutant, *ga1-3*, the floral organs were severely retarded, in particular in the petals and stamens, whereas the forms of the flower’s primordia are not affected [8]. Nevertheless, although the molecular mechanisms of phytohormones have been extensively studied in multiple organs of model and non-model plants, the involvements of the differently structured florets in the single capitulum are not yet understood.

Recently, the transcriptome of *G. hybrida* was established from the high-throughput sequencing of expressed cDNAs, and the expression profiling of different tissues at various flower stages was also constructed using a microarray strategy [3], [9]. After integrating the information obtained from these expression profiles and thoroughly researching a few MADS-box paralogs, an ABCDE model of *G. hybrida* floral development was constructed in 2006 [1], [10], [11]. However, because this profiling was restricted by sequencing technology, only 16,994 *Gerbera* expressed sequence tags (ESTs) have been yielded. Currently, there are only 487 nucleotide sequences, 17,000 ESTs and 339 proteins from *G. hybrida* in the NCBI public databases. Only a few segments related to phytohormonic biosynthesis and signal transduction were identified. Because probes designed for microarray chips are based on gene annotations, expression profiles can only cover small portions of genes and do not reflect the full view of the species’ genome [12]. Despite significant advances in *G. hybrida* genomic research, the old-fashioned, hybridization-based methods, such as microarray, are gradually being replaced by the next generation sequencing technology because of their lack of sensitivity and accuracy for large-scaled genomic information, except in arabidopsis.

RNA-seq (Deep-sequencing of cDNA) provides a massive, unbiased approach to systematically define the transcriptome of an organism and is more sensitive than microarray hybridization [13]. RNA-seq has been successfully used for transcript annotation and/or SNP discovery in many plant species to identify many novel transcript regions [14], [15], [16], [17], [18]. Asteraceae is the largest plant family in the world which contains greater than 24,000 species [19]. In 2010, a project supported by multiple government agencies aiming to sequence the *Helianthus annuus* (sunflower) genome was launched in Canada. However, to date, the entire genome of a single Asteraceae species has not been published. This lack of genomic information results in an incomplete annotation of the transcriptome and limits the study of molecular mechanism within the family. In addition, researchers are overburdened by the vast amount of information obtained from next generation sequencing technologies, such as RNA-seq. From an academic and practical standpoint, it is highly desirable to find suitable ways to analyze the gene messages acquired from transcriptome annotation.

Herein, we reported a massive transcriptome annotation of the *G. hybrid* ray florets using an Illumina HiSeq™ 2000 platform, and we attempted to systematically arrange the series of transcripts associated with gibberellin metabolism and signal transduction. This technological application helped us to evaluate the present theories and comprehend the crucial roles of phytohormones in floral development.

## Results and Discussion

### RNA-seq Using Illumina Platform and Assembly of UniGenes

**Figure 1:**
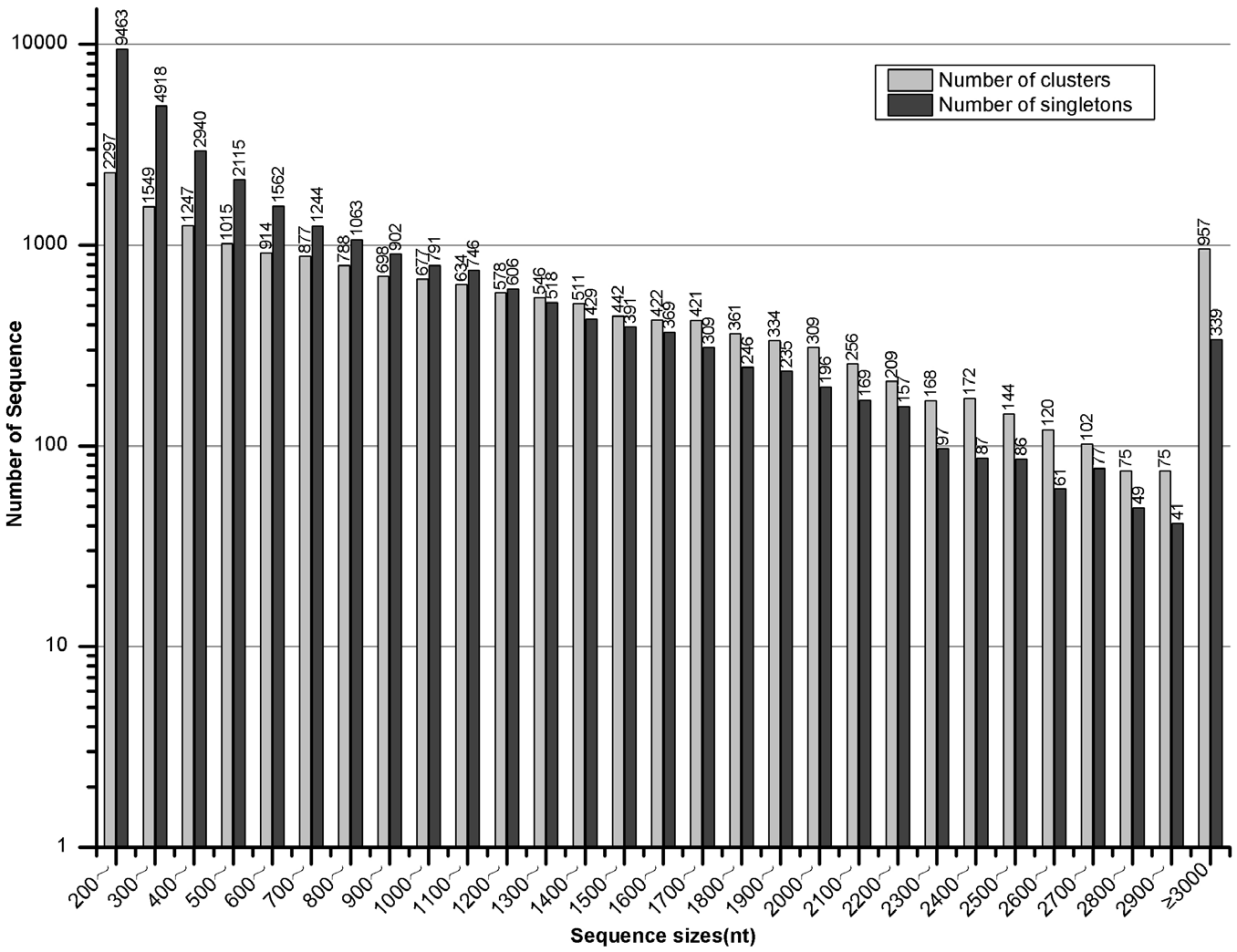
Statistics of Contig and UniGene assembly qualities. All sizes of the Contigs and UniGenes were calculated.

A total of 72,688,546 reads were generated using the Illumina platform. After filtering low-quality reads, 65,561,528 clean reads with 5,900,537,520 nt were selected for further assembly. These short reads were *de novo* assembled into 91,193 Contigs by paired-end joining and gap-filling using Trinity software [20]. The average length of these Contigs was 414 nt with the N50 equaling 868 nt, ranging from 100 nt to >3,000 nt (Table 1, Figure 1). Afterward, the Contigs were connected until neither end was extended. A total of 47,104 UniGenes were obtained with an average length of 845 nt and a final N50 equal to 1321 nt (Table 1). The size distributions demonstrated that the lengths of 13,512 (28.68% of the total 47,104 UniGenes) UniGenes were greater than 1000 nt (Figure 1).

**Table 1:** Summary of the transcriptome of Gerbera hybrida ray florets.

	Total Number	Total Nucleotides (nt)	Mean Length (nt)	N50
Total number of reads	72,688,546	–	–	–
Total number of clean reads	65,561,528	5,900,537,520	–	–
Total number of Contigs	91,193	–	414	868
Total number of UniGenes	47,104	–	845	1321

The assembly software Trinity is excellent for reconstruction and highly sensitive; it can cover more full-length transcripts across a broad range of expression levels than other *de novo* transcriptome assemblers [20], [21]. To evaluate the accuracy of the assembled sequences, we implemented six published genes and eight unpublished genes from the same cultivar that were cloned by our laboratory (% identity≥99%) to compare them to the transcriptome database using a local BLASTN. The results from this comparison study of published genes yielded only one gene with zero base mismatches, three with one base mismatch and two with four and six base mismatches (Table S1). For both the *GASA-like protein* and *Myb10*, the matched sequences were broken into two sections, most likely caused by shearing differences. The comparison results were better for the unpublished, recently cloned genes than for the published clones. Among the eight cloned genes, three of them had zero base mismatches, four had one base mismatch and only one had two base mismatches (data not shown). Excluding the limitations of the sequencing technique and the probability of human errors, we believe that the assembly results are considerably reliable.

### Annotation Functions and Pathways of UniGenes

**Figure 2:**
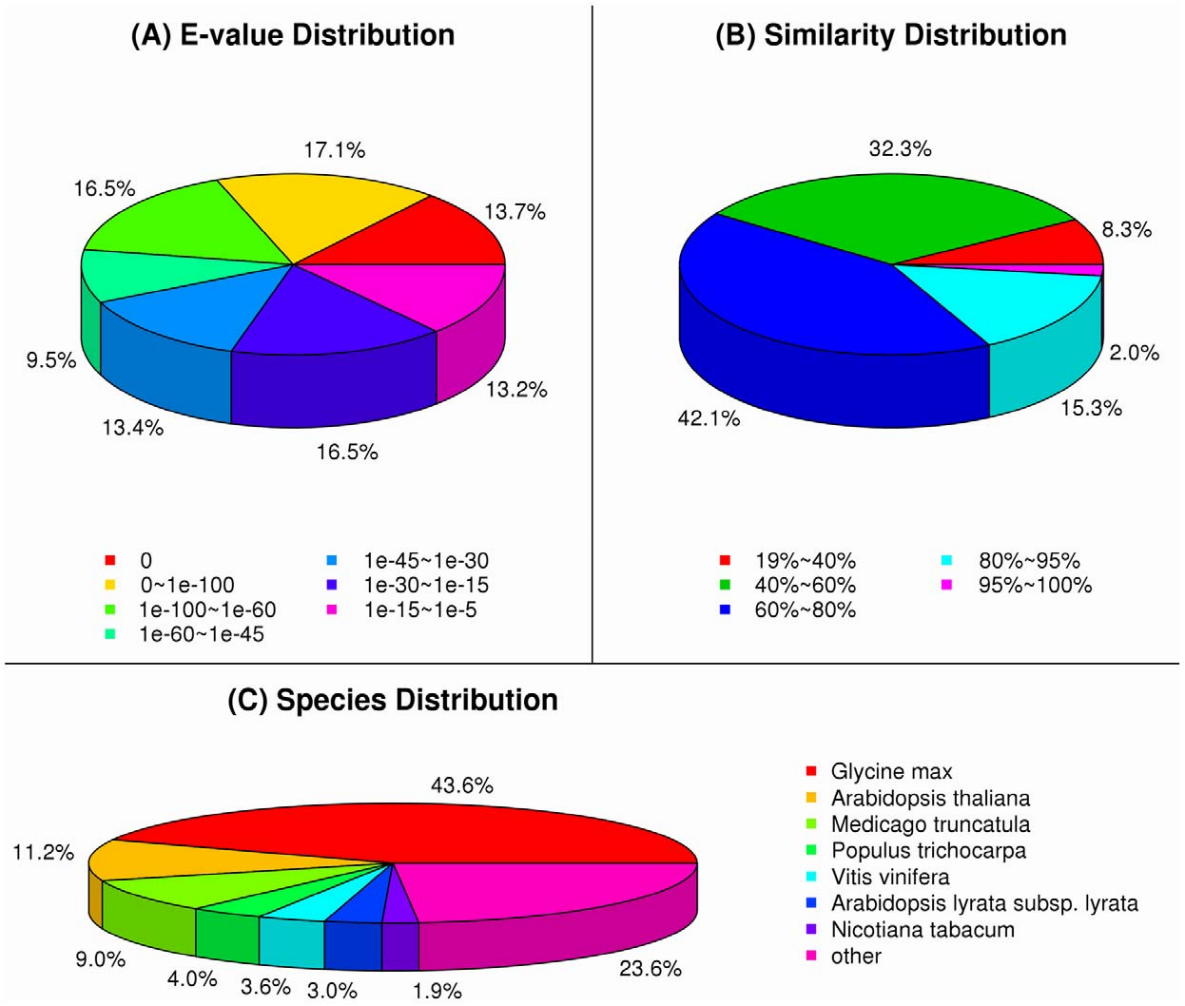
Figures of Nr classification. (A) E-value distribution. (B) Similarity distribution. (C) Species distribution.

After 223 sequences were excluded for exogenous contaminated species in all 47,104 assembled sequences, a total of 36,693 (78.27% of all 46,881 cleaned UniGenes) UniGenes were annotated using BLASTX against Nr with a cut-off E-value of 1e-5 (Table 2, Figure 2A). The E-value distributions of the UniGenes in the Nr database showed that approximately 30% of the UniGenes have strong similarity (smaller than 1e-100), whereas the remaining 70% of the sequences ranged from 1e-5 to 1e-100. The ratios of the similarity distributions demonstrated that 17.3% of the sequences have a similarity over 80%, and 82.7% of the sequences have a similarity ranging from 19% to 80% (Figure 2B). The species distributions for the best match from each sequence are shown in Figure 2C. We hypothesized that the gerbera transcriptome sequences would have close matches to Solanaceae because of their close phylogenic relationship. Notably, the results revealed that the sequences of gerbera ray florets showed more similarity to Leguminous than to other families, with a 43.5% match to *Glycine max*, a 9.0% match to *Medicago truncatula*, and only a 1.9% match to *Nicotiana tabacum* (Figure 2C). We not only compared seven model plants to the entire genomes but also located the transcriptome sequences of several typical Asteraceae species’ ESTs, including *G. hybrida*, *Lactuca sativa* (lettuce), *Chrysanthemum*×*morifolium* (dendranthema), Z*innia elegans* (zinnia), *Artemisia tridentata* (artemisia), *Helianthus annuus* (sunflower), *Carthamus tinctorius* (carthamus) and *Senecio scandens* (senecio). The annotation data showed that 23.8% of the sequences match the reference databases of Asteraceae species. In addition, 28,245 (60.25% of all cleaned UniGenes) UniGenes were further annotated using BLASTN against Nt under identical parameters.

**Table 2:** Summary of the annotations of Gerber hybrida ray floret UniGenes.

	Number of blasted UniGenes	Ratio
All UniGenes	47,104	–
Unigenes of exogenous contaminated species	223	–
All cleaned UniGenes	46,881	100.00%
UniGenes blasted against plant Nr	36,693	78.27%
UniGenes blasted against plant Nt	28,245	60.25%
UniGenes blasted against Swiss-Prot	23,040	49.15%
UniGenes blasted against KEGG	20,375	43.46%
UniGenes blasted against GO	15,721	33.53%
UniGenes blasted against COG	13,239	28.23%
All annotated UniGenes	37,389	79.75%

**Figure 3:**
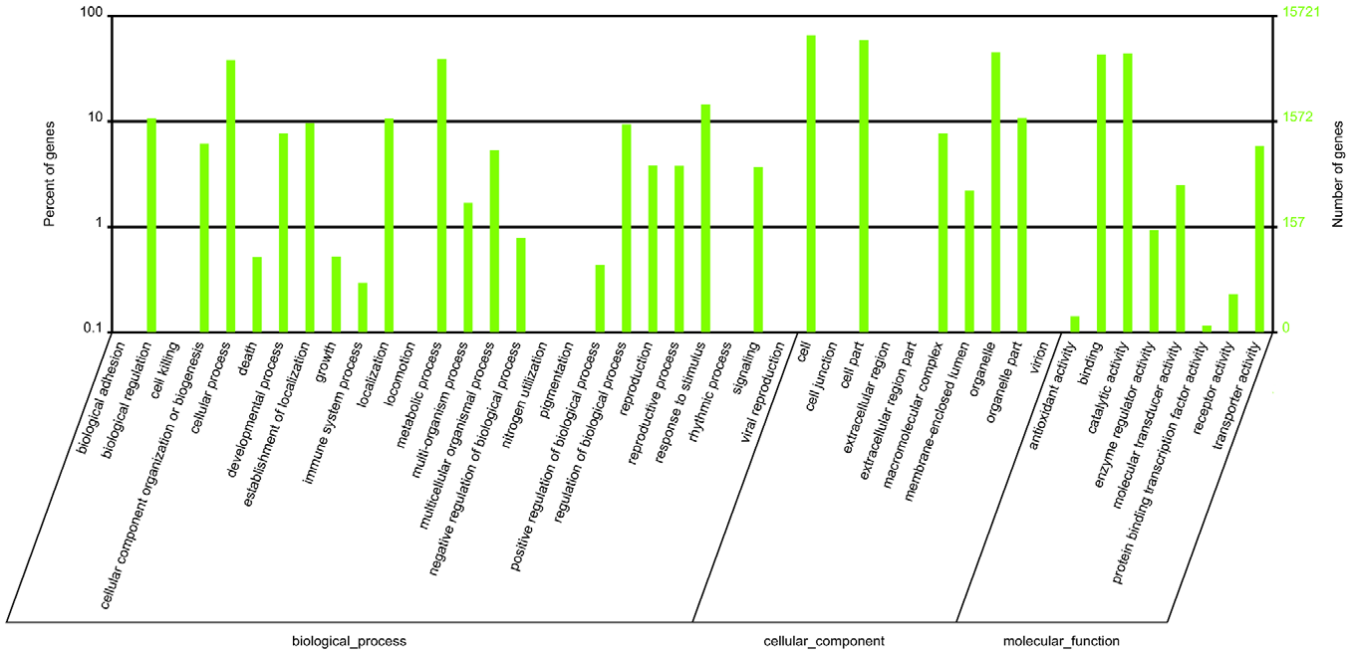
GO categories of the UniGenes. The UniGenes were annotated in three categories: biological processes, cellular components and molecular functions.

**Figure 4:**
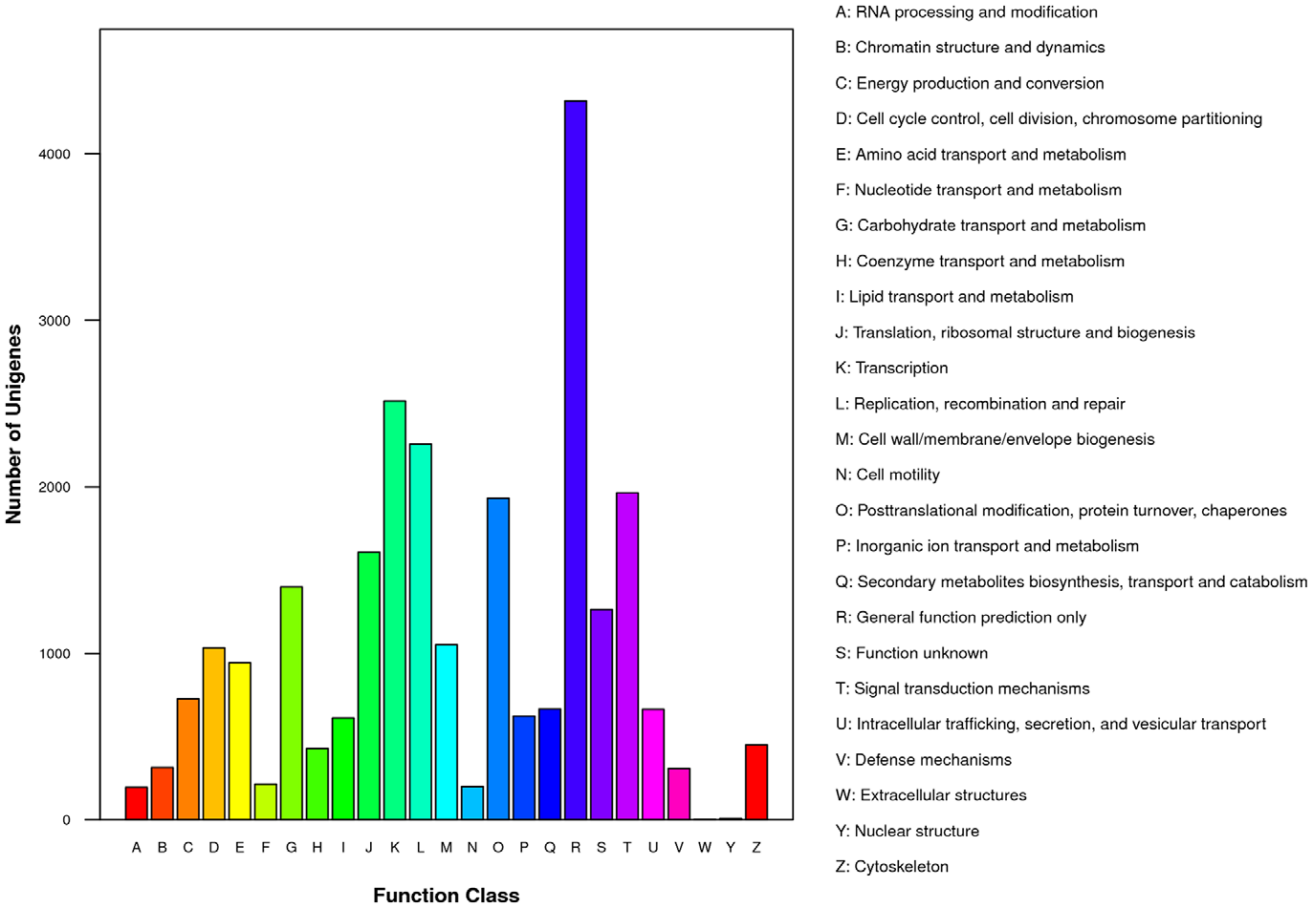
COG function classification of UniGenes.

UniGenes were also aligned to other databases, including 23,040 (49.15% of all cleaned UniGenes) sequences in Swiss-Prot, 20,375 (43.46% of all cleaned UniGenes) sequences in KEGG, 15,721 (33.53% of all cleaned UniGenes) sequences in GO and 13,239 (28.23% of all cleaned UniGenes) sequences in Cluster of Orthologous Group of protein (COG) with the same identical cut-off E-value to supplement the annotations and functions. In all, 37,389 annotated transcripts were identified, representing approximately 79.75% of all cleaned UniGenes (Table 2). The distribution of gene functions in GO from the macro levels was compartmentalized, as shown in Figure 3. In each category of GO classification, the terms “cellular process and metabolic process”, “cell, cell part and organelle” and “binding and catalytic activity” were dominant (Figure 3). Similarly, putative proteins annotated by COG were classified into 25 molecular families, and the top category was “general function prediction by” (Figure 4).

A total of 20,375 UniGenes were annotated in KEGG and located to 126 known KEGG pathways (data not shown). By mapping enzyme commission (EC) numbers against the KEGG database, many transcripts were found that may be involved in the metabolism and signal transduction of eight critical phytohormones, including auxin (AUX), cytokinin (zeatin), GA, ethylene (ET), brassinosteroid (BR), abscisic acid (ABA), jasmonic acid (JA) and salicylic acid (SA). These pathways are involved in tryptophan metabolism, zeatin biosynthesis, diterpenoid biosynthesis, cysteine and methionine metabolism, brassinosteroid biosynthesis, carotenoid biosynthesis, α-linolenic acid metabolism, phenylalanine metabolism and plant hormone signal transduction. Among them, the metabolisms of zeatin, GA, BR and ABA are all derived from primary terpenoid backbone biosynthesis. In particular, GAs play important roles in the development of reproductive organs by modulating floral initiation, promoting floral expansion, enhancing stamen elongation to release and germinate pollens and subsequently stimulating pollen tube growth [8], [22], [23]. Because of these concerns, we have listed the transcripts with putative functions for controlling the GA metabolic pathway, controlling signal transduction, and interacting with other hormonal signal transduction in the next sections.

### Candidate Genes in GA Metabolism

**Figure 5:**
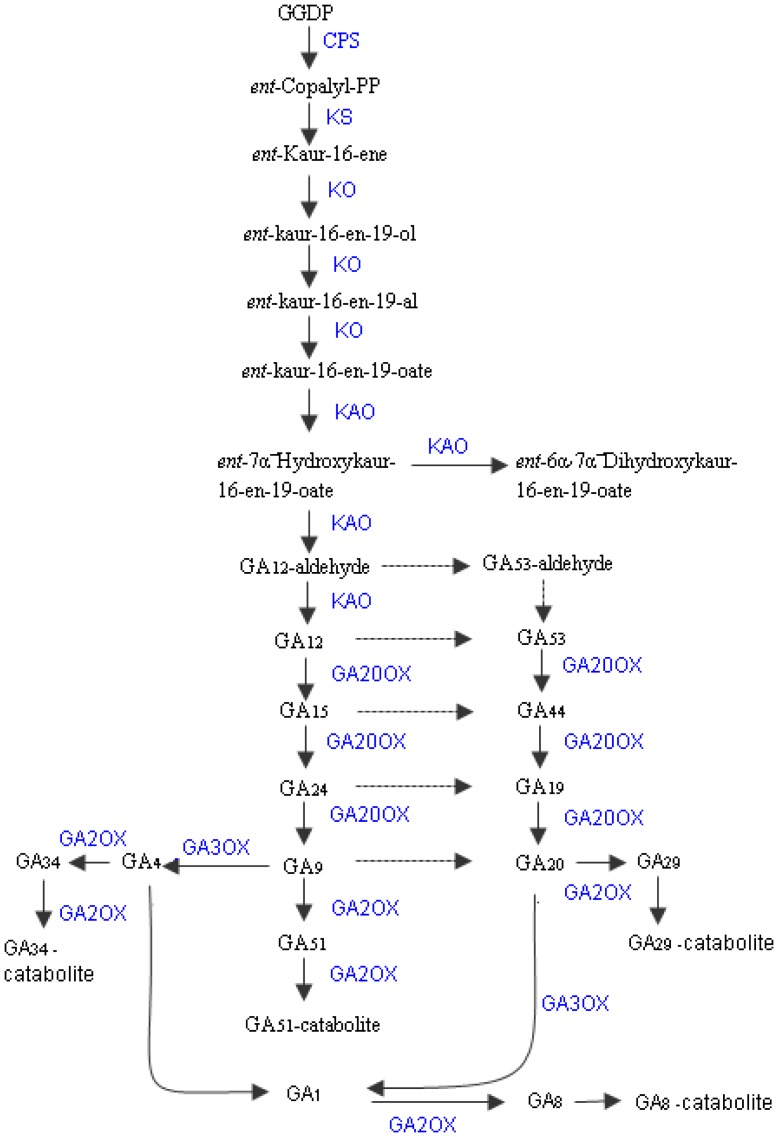
Proposed pathways of GA metabolism in *G. hybrida* ray florets (derived from KEGG map00904, with modification). The compounds are noted in black, and the identified enzymes in *G. hybrida* florets are noted in blue.

Generally, seven enzymes in GA metabolism in plants have been thoroughly researched, including *ent*-copalyl-diphosphate (CPS), *ent*-kaurene synthase (KS), *ent*-kaurene oxidase (KO), *ent*-kaurenoic acid hydroxylase(KAO), GA 20-oxidase (GA20ox), GA 3-oxidase (GA3ox) and GA 2-oxidase (GA2ox) [24], [25], [26], [27], [28], [29]. The synthesis of GAs begins with geranylgeranyl diphosphate (GGDP) catalysis by CPS and KS (Figure 5). One *CPS* and four *KS* transcripts were identified (Table 3 and Table S2), and the *KS* transcripts were located in plastids according to their GO cellular components. KO and KAO belong to the cytochrome P450 monooxygenase family, which catalyzes the sequential oxidation steps (Figure 5). Both *KO* and *KAO* were identified as three transcripts (Table 3, Table S2). The RT-PCR results demonstrated that *KAO* was relatively over-expressed at stage 1 and stage 2. Although these genes are involved in GA synthesis, they do not play pivotal roles in the regulation of GA biosynthesis. *SCARECROW-LIKE 3* (*SCL3*) belongs to the GRAS family that directly interacts with DELLA. The *SCL3*-deficient mutant up-regulates the expression of *GA20ox1*, *GA20ox2*, *GA20ox3* and *GA3ox1*, instead of altering the expression of *KS*, *KO* and *KAO* [30]. Research studies on wheat also indicate that *TaCPS*, *TaKS*, *TaKO* and *TaKAO* do not have obvious feedback regulation [28]. These results imply that the enzymes at the later steps of GA metabolism more precisely regulate concentrations of bioactive GA.

**Table 3:** Statistics of GA metabolism related genes in G. hybrida ray florets.

Symbol	Number of EC	Count of transcripts	Distribution of corresponding hits by local BLASTN			
			*G. hybrida* ‘Terra Regina’	*A. annya*	*C. tinctorius*	*H. annuus*
CPS	5.5.1.13	1	–	5	–	–
KS	4.2.3.19	4	–	5	–	–
KO	1.14.13.78	3	–	1	44	14
KAO	1.14.13.79	3	–	–	–	5
GA20ox	1.14.11.12	5	–	1	5	8
GA3ox	1.14.11.15	5	1	6	18	12
GA2ox	1.14.11.13	6	–	8	28	–

**Figure 6:**
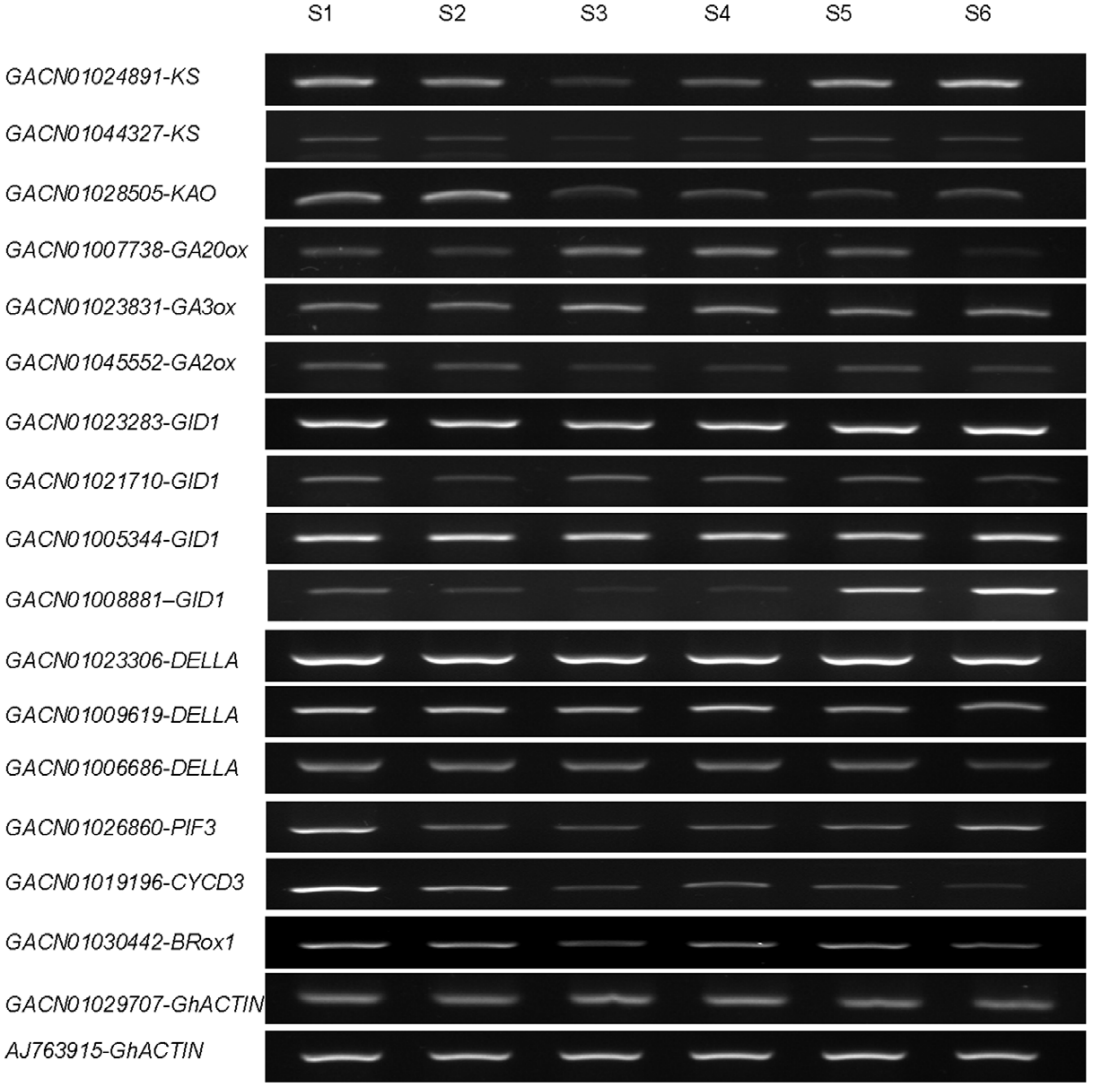
RT-PCR for some candidate genes in stage 1 (S1) to stage 6 (S6). RT-PCR analysis was repeated on three independent samples and the representative ethidium bromide gel pictures are shown. Primer pairs for each distinct gene are listed in Supplemental table S5. *GACN01029707* and *AJ763915* were used as the normalization controls.

GA20ox and GA3ox are the key enzymes in GA biosynthesis. Both *GA20ox* and *GA3ox* were identified in five transcripts of *G. hybrida* ray florets transcriptome (Table 3, Table S2). In Arabidopsis, *GA3ox1* and *GA3ox3* in stamen filaments and flower receptacles play major roles in anther development and petal development [31], [32]. Emasculation of stamens in petunia (*Petunia hybrida*) arrests corolla growth, which can be rescued by exogenous GAs [33]. The expression of *GA20ox* displayed slight up-regulation at stage 3 and stage 4, which was possibly caused by the petal elongation and expansion (Figure 6). Because of the aborted stamens in ray florets, we speculated that the bioactive GAs are transported from the tiny receptacle under every sole floret or from other places in *G. hybrida* to the petal to promote its development. However, why the hermaphrodite disc florets located at the same capitulum have extremely short instead of long petals remains unclear.

An overdose of GA results in a damaged flower opening and fruit ripening [34]. GA2ox as the major deactivation enzyme are essential for precisely sustaining the optimal bioactive GA concentration (Figure 5). Six transcripts of *GA2ox* were identified in our experiment. The expression of *GA2ox* displayed tiny up-regulation at stage 1 and stage 2. We selected the paralogs from four EST databases, *G. hybrida* ‘Terra Regina’, *A. annya*, *C. tinctorius* and *H. annuus* using local BLASTN. The results demonstrated that only one hit of *GA3ox* was found in *G. hybrida* ‘Terra Regina’ and a different number of hits were filtered from the other three species (Table 3). Thus, further study is required on the genes associated with GA biosynthesis.

### Candidate Genes Related to GA Signal Transduction

**Figure 7:**
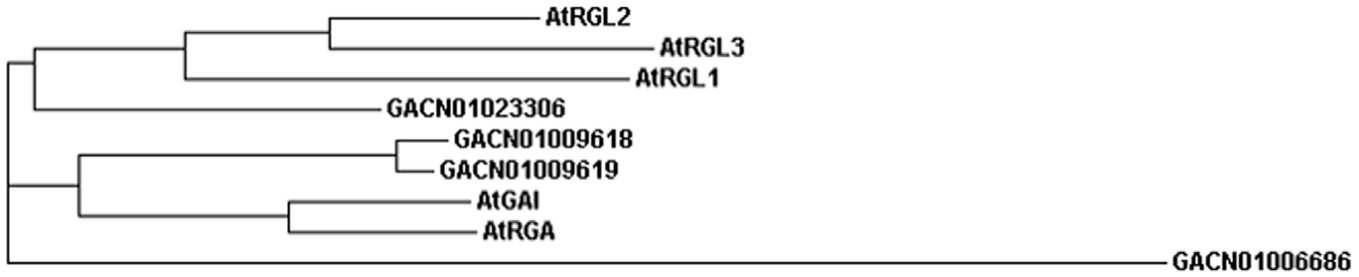
Phylogram tree of five AtDELLA proteins and four putative DELLA proteins in *G. hybrida* using ClustalW2.

The elements of signal transduction in GA, receptor GID1 and inhibitor DELLA proteins, have been studied extensively [35], [36]. The famous *GID1* mutant genotype in rice is extremely dwarfed and completely insensitive to GAs [36]. DELLA proteins occupy the pivotal position, linking the GID1 receptor and its downstream genes. Structures of DELLA proteins contain a characteristic conserved C-terminal GRAS (GAI, RGA and SCR) and a variable N-terminal [37]. Nine transcripts of *GID1* and five *DELLA* transcripts were annotated in the KEGG pathway through rigorous criteria (Table 4, Table S2). In Arabidopsis, there are a total of five DELLA proteins. RGA and RGL2 play more notable roles than other DELLAs in floral development, and their function is enhanced by RGL1 [8], [38]. We selected four candidate transcripts along with the DELLA and GRAS domain comparison with AtDELLAs to construct the phylogram tree using ClustalW2 online (Figure 7). These sequences were aligned first by Clustal Omega. In the tree, GACN01023306 proteins were more highly similar to AtRGLs, whereas GACN01009618 and GACN01009619 were clustered with AtGAI and AtRGA. In addition, GACN01006686 possessed the longest distance from the AtDELLA proteins.

**Table 4:** Statistics of GA signal transduction genes in G. hybrida ray floret.

	Receptor	Transcript factor	Number of transcripts	Distribution of corresponding hits by local BLASTN			
				*G. hybrida* ‘Terra Regina’	*A. annya*	*C. tinctorius*	*H. annuus*
Gibberellin	*GID1*		9	1	71	11	3
		*DELLA*	5	–	58	1	53
		*GID2*	6	–	2	5	
Several downstream genes in GA signal		*PIF1*	1	–	1	–	–
		*PIF3*	1	–	5	–	3
		*BR6ox1*	1	–	7	8	–
		*CYCD3*	8	–	169	110	22

DELLAs suppress the expression of downstream genes by binding domains in their promoters and they are degraded by the 26S proteosome [39], [40]. RT-PCR results demonstrated that the expression of the three *DELLA* transcripts was almost unchanged from stage 1 to stage 6, and only one *GID1* showed higher expression at stage 5 and stage 6 (Figure 6). GID2 in rice and SLEEPY (SLY1) with its homolog SLY2 in Arabidopsis belong to the F-box protein family, which is also involved in the degradation of DELLAs [41]. We identified six transcripts of *GID2* in the transcriptome database (Table 4, Table S2).

Many downstream DELLA genes are involved in regulating the signal transduction of GAs. Compared to GA-regulated genes in Arabidopsis [42], 14 target genes responded directly to both GA and DELLA after short-term GA treatment and were filtered using microarray chips combined with real-time RT-PCR analysis [35]. We could exclusively identify a few matched homologs encoding *GA20ox*, *GA3ox* and *GID1*, which were mentioned in the previous section (Figure 4). We could only identify a few matched homologs probably because of the discrepancies in species and incomplete annotation in our transcriptome. However, there remains doubt that *GA20ox* and *GA3ox* are not DELLA primary targets because binding between the RGA domain and their promoters has been undetected, even though the transcript levels of the two genes change a short time after DELLAs degradation [35], [43].

In addition, several other transcript factors are directly regulated by the DELLA’s binding domain, such as the *PHYTOCHROME-INTERACTING FACTORS* (*PIF*s) from the bHLH family. DELLAs bind to PIF3 and PIF4 to repress hypocotyl expansion or chloroplast development, linked to the light response and the circadian clock [44], [45], [46]. PIF1 (PIL5) delays germination in the dark not only by repressing the expression of *GA3ox* but also by promoting the expression of *GA2ox* to regulate endogenous GA levels and the expression of two DELLAs, GAI and RGA [47]. We only identified one PIF1 or PIF3 in our transcriptome (Table 4, Table S2). GO annotation demonstrated that these genes possess the ability to bind nucleic acids as homologous genes and that these genes are located in intracellular membrane-bound organelles. PIF3 was up-regulated at stage 1 and stage 6 compared to other stages (Figure 6).

Signals of the other seven phytohormones were also involved in the development of ray florets. The genes in the pathway marked at the KEGG map04075, which encode as phytohormone signal transduction, are statistically listed in Table S3 and Table S4. These results imply that the growth of the ray floret involves integral modulations that are controlled by the endogenous phytohormones and exogenous stimuli. As the synthesis of GA, ABA, cytokinin and BR is all derived from pathways of terpenoid backbone synthesis by utilizing common terpoid precursors, the four phytohormones may influence one another through several important roles. One of the RING-H2 ubiquitin E3 ligases, *XERICO*, plays a role not only in ABA metabolism but also as a target of DELLA in GA response [35], [48]. Unfortunately, we could not identify perfectly matched transcripts of *XERICO*. The gene, *Brassinosteroid-6-oxidase2* (*BR6ox2*), which encodes the cytochrome P450 enzyme to catalyze the last step of BR biosynthesis, is also down-regulated by GA early response [35], [49]. Only one homologous transcript of *BR6ox1* was identified in the transcriptome. The expression of *CYCLIN D3* (*CYCD3*) is modulated by cytokinin levels to influence cell division and determine cell number in developing organs [50]. Overexpression of *CYCD3* can rescue the growth of *ga1-3* plants [51]. Several results indicate that cell proliferation under GA stimuli may affect cytokinin signal transduction by controlling the *CYCD* gene family. We identified eight transcripts of *CYCD3* (Table 4, Table S2). RT-PCR results demonstrated that *CYCD3* (accession ID GACN01019196) was obviously up-regulated at the stage 1 and stage 2 (Figure 6). As CYCD3 is the mark gene for cell division, this result is consistent with fact that the ray florets are in cell division in early stages as well [9]. Because we did not obtain the *BR6ox2* and detect the cytokinin level, we cannot conclude the possibility that the variation of *CYCD3* is not directly influenced by the phytohormone. These results indicate that the development of the *G. hybrida* ray floral is a network that is controlled by the dynamic homeostasis of multiple phytohormones in response to environmental factors.

The transcriptome provides useful information for searching homologs to research their functions. Compared to the numerous hits found for the other three species, just only one hit of *GID1* was found in the signal transduction genes in *G. hybrida* ‘Terra Regina’ (Table 4). Similarly, in the GA metabolism pathway, one hit of *GA3ox* was found in the same EST database. Considering these results, our transcriptome of *G. hybrida* ray florets is a useful and essential supplement to the gene database.

### Conclusions

In this study, we compiled a massive-pool transcriptome from a non-model species for further molecular research, as a whole genome database is currently lacking. This study represents the first application of high-throughput transcriptome profiling of *G. hybrida* ray florets using RNA-seq technology. According to the annotation, we summarized various types of transcripts that encode for putative genes that are involved in GA metabolism, signal transduction and feedback regulation. We also found several putative genes that are associated with the signal transduction in other phytohormones and that are involved in GA modulation. The profile indicated that *G. hybrida* flowers, particularly the ray florets, could potentially be used to study the interaction and regulation of phytohormones. These clues should help to identify the functions of the novel homologous genes using high-throughput sequencing technology.

## Materials and Methods

### Materials and Growth Conditions

*G. hybrida* ‘Shenzhen No. 5′ seedlings were grown under a standard greenhouse (Zengcheng Ornamental Center, Guangzhou) at 26/18°C±2 (day/night) with a relative humidity between 65% and 80%. The development of inflorescence was divided into six stages, as previously described according to the shapes of the ray florets [52]. Compared to the developmental stages in *G. hybrida* ‘Terra Regina’, which are divided into 11 stages by Helariutta [53], the stages we divided in *G. hybrida* ‘Shenzhen No. 5′ correspond to one another, such as stage 1 (S1) corresponding to Helariutta’s stage 2; S2, stage 3; S3, stage 4; S4, stage 5; S5, stage 7; and S6, stage 8. At the S6, our ray florets are fully opened, but the anthers are not visible in the disc florets. We assumed that almost all of the genes related to development are already present before S6 and that the flower has begun senescence at S6. In addition, our major focus was on genes before S6. Therefore, we picked up the ray florets in five sample pools from early S1 to S5 to constitute our cDNA library for the transcriptome of flower growth. All collected fresh samples were snap-frozen immediately in nitrogen and stored at −80°C to extract the total RNA.

### cDNA Library Preparation for Illumina Sequencing

Total RNA was extracted using the TRIZol reagent and an isolation system according to the manufacturer’s protocol and treated with DNase. Both the concentration and integrity of the RNA samples for transcriptome analysis were evaluated with Angilent 2100. Samples with an integrity value greater than 8 were used to construct the cDNA libraries. To avoid losing low-expression transcripts during early envelopment, a pooled RNA sample with early S1, S2, S3, S4 and S5 was mixed at a ratio of 2∶1.25∶1∶1∶1 for cDNA. The cDNA library preparation and sequencing reactions were conducted at the Beijing Genomics Institute (BGI) genomic center, Shenzhen, China (http://www.genomics.cn). The procedure is briefly described below.

Magnetic beads with Oligo(dT) were used to isolate the poly(A)^+^ mRNA after all of the total high quality 20 µg RNA samples were mixed. Fragmentation buffer was added in the presence of divalent cations at 94°C for 5 min to interrupt the mRNA into short fragments of approximately 200 bp. These short fragments were used as templates, and random hexamer-primer was used to synthesize the first-strand cDNA. The second-strand cDNA was synthesized using buffer, dNTPs, RNaseH and DNA polymerase I. The short fragments were purified with a QiaQuick PCR extraction kit and resolved with EB buffer to end reparation and add poly(A)^+^. The suitable fragments (approximately 375 bp) with sequencing adaptors were selected as templates for the PCR amplification based on the agarose gel electrophoresis results. The samples were clustered in flow cells to construct the cDNA library and loaded onto an Illumina HiSeq™ 2000.

### Sequencing, Assembly and Annotation of the Transcriptome

The cDNA library was sequenced from the 90 bp end-paired size using Illumina HiSeq™ 2000 according to the manufacturer’s instructions. The initial base setup and quality filtering of the image data were performed using the default parameters in the Illumina data processing pipeline.

The adaptors, any unknown nucleotides larger than 5% and low-quality reads containing more than 20% bases with Q-values ≤10 were removed after sequencing. Processed reads with an identity value of 95% and coverage length of 180 bp were assembled using Trinity software, which consists of three modules: Inchworm, Chrysalis and Butterfly [20]. The software first combined reads of certain lengths of overlap to form longer fragments called Contigs. Then, the reads were mapped back to the Contigs, which were connected until extended on neither end. The obtained sequences were defined as UniGenes after removing any redundancy. The redundant sequences were set up as the identity of overlapping areas which was arrived at 94% with the lengths greater than 100 bp.

These UniGenes were submitted to protein databases for homolog and annotation comparison by BLASTX algorithm (e-value ≤1e−5), including Nr, Swiss-Prot, KEGG, and COG. The Blast2GO [21] program and WEGO [22] software were used in GO annotation and functional classification. BLASTN was used in the Nt nucleotide database. ESTScan software (http://www.ch.embnet.org/software/ESTScan.html) located the position of the UniGene sequences which were unaligned to the previously mentioned databases [54].To compare the similarities with those genes cloned in our laboratory, we performed by local BLASTN (version 2.2.23). After annotation, the databases were submitted to NCBI. Files containing the row read sequences and their quality scores were accessible from Short Read Archive run accessions (SRR): SRR611397. The assembled sequences (great than 200 bp) have been placed in the Transcriptome Shotgun Assembly Sequence Database (Bioproject: 179026 TSA) with the accession number GACN00000000 (From GACN01000001 to GACN01046881) after excluding the exogenous contaminated sequences.

### Criteria for Screening Candidate Genes during Pathway Analysis

To obtain information regarding the metabolism and signal transduction of the phytohormones, we roughly filtered the transcripts that are involved in these pathways by the corresponding annotation of KEGG and KO ID in the KEGG maps (http://www.genome.jp/dbget/). However, the annotations of one transcript from the Nr, Swiss-Prot and KEGG databases were not always consistent with one another. Therefore, more stringent screening criteria were set up to ensure that the annotations were consistent in at least two different databases. Some sequences with ambiguous annotations were confirmed online by Nr comparison using BLASTX (http://blast.ncbi.nlm.nih.gov/Blast.cgi). To compare the homologs with other species in the Asteraceae, we implemented screening parameters as e-value ≤1e−5 using local BLASTX (version 2.2.23). The multiple alignment analysis of the DELLAs was performed in *G. hybrida* and *Arabidopsis thaliana* to construct the phylogram tree using ClustalW2 (http://www.ebi.ac.uk/Tools/phylogeny/clustalw2_phylogeny/).

### Semi-quantitative RT-PCR Analysis of the Filtered Genes

As the transcriptome was the pooled data with the mixed libraries, semi-quantitative RT-PCR was chosen to detect the expression of the selected genes expression in distinct stages. The candidate genes with lengths greater than 500 bp were initially screened for being designed the primers. Then, in the selected genes, conserved areas were identified using local BLASTN and DNAMAN software to avoid locating primers in there. The sequences with high identities (greater than 90%) to others were excluded for being designed effective primers. The primer pairs in the selected sequences were designed by Primer Primier 6 software and listed at Table S5. The internal reference sequences used two *ACTIN*s (accession number GACN01029707 and AJ763915, respectively). The former sequence was selected from our transcriptome, and the latter was downloaded from the EST database of *G. hybrida* in the Genbank. The FPKM (Fragments Per Kilobase of exon model per Million mapped reads) value of *GACN01029707* is 530 in the transcriptome. In the expression profiling treated with GA for 0 h and 2 h, the values of RPKM (Reads Per Kilobase of exon model per Million mapped reads) were 720 and 665, respectively (data unpublished). As both FPKM and RPKM were over 300, we considered the *GACN01029707* can be used as a housekeeping gene for normalization control. *AJ763915* was assigned for the double confirmation. Total RNA from stage 1 to stage 6 was extracted using identical methods as those mentioned before. cDNA was synthesized with 2.5 µg of RNA with oligo(dT) and reverse transcriptase following the protocol for a 50 µl system (Takara). PCR was performed with a 1 µl template for the PCR reaction at 94°C for 3 min, then 94°C for 30 s, 60°C for 20 s, and 72°C for 40 s with 25 to 30cycles in a 20 µl system.

## Supporting Information

Table S1 **Match results of published genes cloned in our laboratory compared to the transcriptome.**
(XLSX)

Table S2 **Specific genes identified and involved in GA biosynthesis and signal transduction.**
(XLSX)

Table S3 **Statistics of signal transduction genes in other hormones.**
(XLSX)

Table S4 **Specific genes identified and involved in signal transduction in other phytohormones.**
(XLSX)

Table S5 **List of primers used for RT-PCR.**
(XLSX)
